# Epidemiological and Clinical Characteristics of Hospitalized Pediatric Patients with SARS-CoV-2 Infection in Mexico City, Mexico

**DOI:** 10.1155/2022/6780575

**Published:** 2022-04-25

**Authors:** Gustavo Esteban Lugo-Zamudio, Antonio Aguilar-Rojas, Martín Uriel Vázquez-Medina, Antonio Gutiérrez-Ramírez, Ma. Cristina Upton-Alvarado, Patricia Espinoza-Rivas, Gustavo Lagunas-Torres, María Isabel Rojo-Gutiérrez, Gabriela Ibáñez-Cervantes, Cruz Vargas-De-León

**Affiliations:** ^1^Dirección General, Hospital Juárez de México, Ciudad de México, Mexico; ^2^Unidad de Vigilancia Epidemiológica Hospitalaria, Hospital Juárez de México, Ciudad de México, Mexico; ^3^Hospital Universitario de Puebla, Benemérita Universidad Autónoma de Puebla, Puebla, Mexico; ^4^División de Gineco-Pediatría, Hospital Juárez de México, Ciudad de México, Mexico; ^5^División de Atención Al Usuario, Hospital Juárez de México, Ciudad de México, Mexico; ^6^Dirección Médica, Hospital Juárez de México, Ciudad de México, Mexico; ^7^División de Investigación, Hospital Juárez de México, Ciudad de México, Mexico; ^8^Sección de Estudios de Posgrado, Escuela Superior de Medicina, Instituto Politécnico Nacional, Ciudad de México, Mexico

## Abstract

**Background:**

Evidence from across the world suggests that the pediatric population shows different clinical manifestations and has a lower risk of severe presentation of SARS-CoV-2 infection compared to adults. However, Mexico has one of the highest mortality rates in the pediatric population due to SARS-CoV-2 infection. Therefore, our objective was to explore the epidemiological and clinical characteristics associated with a positive confirmatory test in the Mexican pediatric population admitted to a tertiary care hospital in Mexico City.

**Methods:**

Clinical, imaging and laboratory data were retrospectively collected from 121 children hospitalized during the period from March 4^th^, 2020, to August 8^th^, 2021. The patients were identified as suspicious cases according to the guidelines of the General Directorate of Epidemiology of Mexico. Real-time polymerase chain reaction (RT-PCR) tests were used to confirm SARS-CoV-2 infection. Categorical variables were compared using the Chi-square test, and propensity score matching was performed to determine univariate and multivariate odds ratios of the population regarding a positive vs. negative SARS-CoV-2 result.

**Results:**

Of the 121 children, 36 had laboratory-confirmed SARS-CoV-2 infection. The main risk for SARS-CoV-2-associated pediatric hospitalization was contact with a family member with SARS-CoV-2. It was also found that fever and fatigue were statistically significantly associated with a positive SARS-CoV-2 test in multivariate models. Clinical and laboratory data in this Mexican hospitalized pediatric cohort differ from other reports worldwide; the mortality rate (1.6%) of the population studied was higher than that seen in reports from other countries.

**Conclusion:**

Our study found that fever and fatigue at hospital presentation as well as an antecedent exposure to a family member with SARS-CoV-2 infection were important risk factors for SARS-CoV-2 positivity in children at hospital admission.

## 1. Background

Several studies show that children and adolescents are as likely as adults to be infected with SARS-CoV-2; however, evidence from different parts of the world suggests that this population shows different clinical manifestations and has a lower risk of severe presentation of SARS-CoV-2 compared to adults [1–4]. For instance, in the pediatric population, the clinical disease symptoms are usually mild and, like other acute viral respiratory infections, involvement of the lower respiratory tract rarely occurs [5]. Comorbidities have a less well-defined role in the risk of having a severe course of SARS-CoV-2 infection in the pediatric population [6]. Laboratory findings and imaging studies also show different features in the pediatric population; typical findings in adults such as lymphopenia are infrequent in children [7].

The first case of SARS-CoV-2 infection in Mexico was detected on February 27^th^, 2020. Mexico has had a high SARS-CoV-2 infection incidence (43%) and reported mortality (9%) [8]. Regarding the different Mexican states, as of January 22, 2022, Mexico City has accumulated the highest number of cases (1.25 million) and has the highest incidence rate (129.5 per 100,000 inhabitants) [9, 10]. Mexico City also has the highest number of deaths reported at the national level (53,119) [9]. In the case of pediatric patients infected by SARS-CoV-2 in Mexico, as of January 22, 2022, a total of 280,214 of positive cases and 1,057 deaths have been reported nationwide. The highest number of deaths occurred in the group under one year of age [9]. The National Database of the Minister of Health shows that positive cases were predominantly seen in the 15–17 year age group [9], 1.8% of the cases required airway intubation, and the reported mortality was 1.9% [11]. Regarding information on the Mexican states, the largest number of confirmed cases of SARS-CoV-2 in the pediatric population was concentrated in Mexico City, with 103,850 cases. Mexico City also reported the highest number of deaths, with 165 cases [9].

There are very few reports on the Mexican pediatric population hospitalized with SARS-CoV-2 and, in the case of Mexico City, the clinical and epidemiological profile of this population is even less well characterized. Macias-Parra and colleagues studied a cohort (*n* = 86) of pediatric patients hospitalized with SARS-CoV-2 infection at a tertiary hospital in Mexico City, finding that 40% of the patients were >12 years old, 21% manifested severe and critical disease, and the mortality of the cohort was 5% [12]. Oliver-López and colleagues studied a cohort (*n* = 510) from a pediatric referral hospital in Mexico City, including inpatients and outpatients that met the criteria for a suspected case by the Mexican Epidemiological Surveillance Directorate [13]. They evaluated the clinical and epidemiological characteristics associated with a confirmatory test for SARS-CoV-2 infection, finding that a history of contact with a positive case was statistically significantly associated with an increased likelihood of having a positive confirmatory test [13].

The mortality reported in the pediatric population with confirmed SARS-CoV-2 infection in Mexico is one of the highest, both nationally and in the hospitalized population [12, 14–16]. In addition, there is a lack of information about the epidemiological, imaging, and clinical profiles of the pediatric population infected by SARS-CoV-2 in Mexico, especially the population that has been hospitalized [8]. Therefore, our objective was to explore the epidemiological and clinical characteristics (including the CO-RADS scale) of the Mexican pediatric population admitted to a tertiary care hospital in Mexico City that are associated with a positive confirmatory test.

## 2. Methods

A retrospective study was conducted. All pediatric patients admitted to a tertiary referral hospital (Hospital Juarez de Mexico) in Mexico City, Mexico with suspected SARS-CoV-2 infection from March 4^th^, 2020 to August 8^th^, 2021 were studied. Pediatric patients were included if they were <18 years old and met the criteria for a suspected case of SARS-CoV-2 infection. The patients were identified as suspicious cases according to the guidelines for epidemiological surveillance of the General Directorate of Epidemiology of the Mexican Ministry of Health (a suspicious case is defined as a person of any age who in the last 10 days has presented at least one of the following signs or symptoms: cough, dyspnea, fever, or irritability, in association with at least one of the following signs or symptoms: myalgia, arthralgia, odynophagia, chills, chest pain, rhinorrhea, polypnea, anosmia, dysgeusia, or conjunctivitis) [17].

Hospital Juarez de Mexico is a third-level hospital and is considered a specialty referral hospital. It is a public hospital that belongs to the Mexican Ministry of Health and serves the general population. In the case of care for patients suspected of SARS-CoV-2 infection, a total of 3,451 patients were treated in the emergency department in 2020, of whom 1,394 were hospitalized and 701 died. The hospital has a pediatric intensive care unit that has eight pediatric ventilators. During the pandemic period, institutional protocols were established for early admission of the pediatric population with SARS-CoV-2 and, according to official reports, the unit was never overcrowded. In addition, during the study period, the hospital had six beds in the pediatric respiratory emergency department for patients suspected of having SARS-CoV-2 infection [17–19].

SARS-CoV-2 infection was confirmed by RT-PCR of a nasopharyngeal swab. SARS-CoV-2 detection was performed using specific primers and probes with the Super-Script III Platinum One-step qRT-PCR System (catalog: 12574035; Invitrogen, Carlsbad, California, USA) on the CFX96 Real Time PCR Detection System (Bio Rad, California, USA). Patients positive for another viral agent as the causative agent of their symptomatology were excluded. Patients were classified as SARS-CoV-2 positive or negative based on the RT-PCR results.

### 2.1. Procedures

Sociodemographic data such as age, sex, body mass index (BMI), symptoms, comorbidities, history of exposure to SARS-CoV-2 infection from a family member (defined as contact at less than 1.5 meters without the use of masks, in the transmission period, with a family member who had a positive RT-PCR test for SARS-CoV-2 infection; the transmission period was considered to be between 2 days before the onset of symptoms and 10 days after [20, 21]), ABO blood group, vaccination scheme (complete Mexican vaccination scheme [22], including SABIN (for polio), BCG (for TB), pentavalent (for diphtheria, pertussis, tetanus, polio, and *Haemophilus influenzae B*), DPT (diphtheria, pertussis, and tetanus), SRP (for measles, rubella, and mumps), SR (for rubella and measles), TD (for tetanus) and the hepatitis B vaccine), outcomes (length of hospital stay, deaths, and intubations), the results of laboratory tests (blood biometry and blood gas test), and CT scan results (including the CO-RADS scale, performed independently by two radiologists according to previous studies [23, 24]) were obtained from electronic clinical records. Normal reference ranges for leukocytes and lymphocytes were adjusted for age according to the reference range values for the pediatric population [25]. Vital sign reference cut-off values for the pediatric population were adjusted for age [26].

The CO-RADS scale is a standardized grading system that evaluates CT findings for patients with suspected SARS-CoV-2 infection. Based on the level of suspicion of SARS-CoV-2 infection, the scale is graded into 6 levels: CO-RADS 1—normal thorax, CO-RADS 2—low, CO-RADS 3—indeterminate, CO-RADS 4—high, CO-RADS 5—very high, and CO-RADS 6—confirmed by RT-PCR [23, 24].

### 2.2. Patient Discharge

The criteria for discharge of pediatric patients from this hospital were normal body temperature for 3 days, two negative RT-PCR results at 24-hour intervals, and resolution of all clinical symptoms.

### 2.3. Statistical Analysis

The data are presented as the mean (standard deviation, SD) and counts (percentage) for numerical and categorical variables, respectively. According to the RT-PCR tests of the patients, two groups were formed (SARS-CoV-2 positive and SARS-CoV-2 negative). Categorical variables were compared using the Chi-square test with or without Yates' corrections. A one-sample exact test was performed to compare the proportion of a symptom versus a reference proportion. The reference proportion of each symptom was obtained from the 2019 meta-analysis by Cui et al. [27]. The binom test function from the *R* “stats” library [28] was used for the analysis.

Propensity score matching (PSM) was performed to determine univariate and multivariate odds ratios (OR) to quantify the increased likelihood of SARS-CoV-2 positivity seen with the clinical features and hematological parameters that showed statistically significant associations with a positive test for SARS-CoV-2 in the univariate analysis. PSM weights were obtained by adjusting the variables age, sex, and comorbidities, thereby minimizing bias and improving risk estimates of SARS-CoV-2 positivity. The covariate balancing propensity score (CBPS) method was used and estimated with the average treatment effect (ATT). The quality of the PSM was checked by comparing the standard mean difference before and after the PSM. PSM analysis was performed using the “weightit” function of the *R* “Weightit” library [29]. We checked the goodness-of-fit of the logistic models with the Hosmer–Lemeshow test. *P* values <0.05 were considered statistically significant. Analyses were performed using IBM Statistics SPSS 21 and *R* software, version 3.4.4, whereas forest plots were made in GraphPad Prism 8.4.0.

### 2.4. Ethical Considerations

This study was approved (Reg. 030/21I) by the research committee of the Hospital Juarez de Mexico (HJM). The present study complied with the basic principles of human research following the Declaration of Helsinki of the Medical Association. An anonymized version of this dataset was used to process confidential patient data without explicit patient consent. All methods were conducted in accordance with relevant guidelines and regulations including the good clinical practice guidelines of COFEPRIS in Mexico.

## 3. Results

From January 2020 to June 2021, Hospital Juarez de Mexico treated 182 pediatric patients with respiratory emergencies and 4,876 pediatric patients with nonrespiratory emergencies [17, 19].

**Figure 1:**
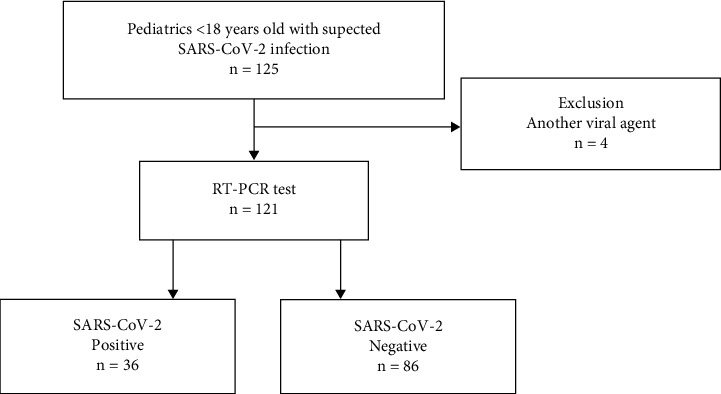
Study flow diagram.

A total of 121 children were seen at Hospital Juarez de Mexico between March 4^th^, 2020 and August 8^th^, 2021 with suspected SARS-CoV-2 infection according to the guidelines for epidemiological surveillance of the General Directorate of Epidemiology of the Mexican Ministry of Health (Figure 1).

Of the children with suspected SARS-CoV-2 infection, 36 of them tested positive for SARS-CoV-2 infection. Of those 44% were boys, 5.6% were in the >15 age range, and 44.4% were between the ages of 11 and 14 years old. There was an association between contact with a confirmed positive family member and a positive SARS-CoV-2 test (*P*=0.01). In addition, 22.2% of the SARS-CoV-2 positive children had contact with a confirmed positive family member.

An association was found between CO-RADS and a SARS-CoV-2 positive test (*P*=0.003). Overall, 71.4% of positive cases were diagnosed with CO-RADS 5, and 57.1% of negatives were diagnosed with CO-RADS 1. In total, 73% of positive patients had undergone a complete vaccination scheme. No association was found between the completion of the vaccination scheme and a SARS-CoV-2 positive test (*P*=0.1). No patient had received the SARS-CoV-2 vaccine.

Obesity was the only comorbidity that was associated with a SARS-CoV-2 positive test (*P*=0.03) in the univariate analysis; asthma, immunosuppression, cancer, hypertension, and allergies were not found to be associated. No association was found with the ABO blood type. In the laboratory evaluation, 72% of the positive patients had lymphopenia and seven (38.9%) had leukopenia (Table 1).

The difference in frequencies of fever (*P*=0.01) and fatigue (*P*=0.03) between SARS-CoV-2 positive and SARS-CoV-2 negative patients was statistically significant in the univariate analysis (Table 2). Thirty-three (91.7%) positive patients had fever, and 60 (70.6%) negative patients had this symptom. In addition, eight (22.2%) and seven (8.2%) of the positive and negative patients experienced fatigue, respectively.

We compared the percentages of symptoms of positive patients against the percentages obtained in the meta-analysis of Cui [25]. We found that the percentages of the symptoms such as fever, sore throat, rhinorrhea, tachypnea, diarrhea, and vomiting were significantly different from the reference percentages of the meta-analysis (Table 3).

**Figure 2:**
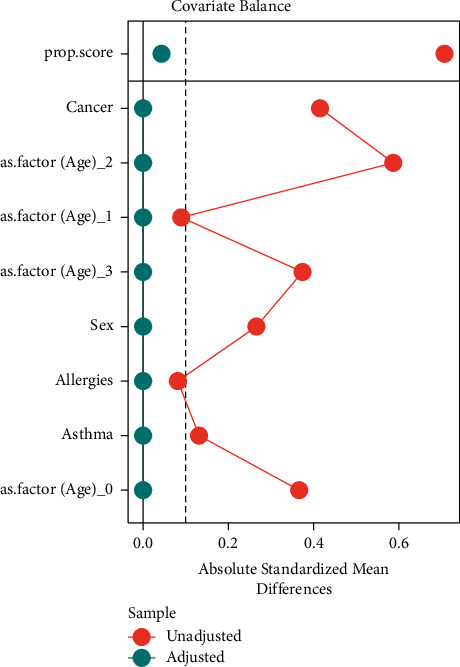
Comparison of standard mean difference before and after PSM.

Figure 2 shows the balance of covariates before and after propensity score matching for COVID-19 positive and negative patients; the plot shows the standardization for comparability.

**Figure 3:**
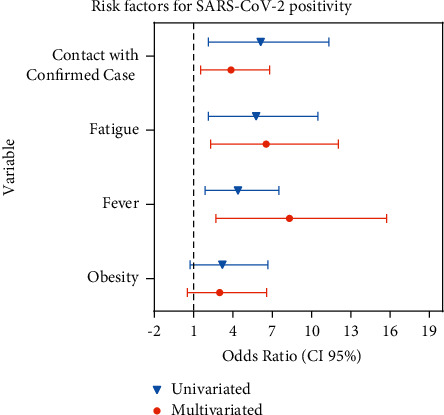
Forest plot of the univariate and multivariate weighted odds ratios with propensity score matching of the risk factors for SARS-CoV-2 positivity.

The results of simple and multivariate logistic regression adjusted with PSM weights are reported in Figure 3. In the multivariate analysis, fever was a 6.51-fold risk factor (multivariate OR, 95% CI: 2.70–15.73, *P*=0.002) compared to negative patients, and fatigue was a 5.26-fold risk factor (multivariate OR, 95% CI: 2.29–12.06, *P* < 0.001). Contact with a confirmed case led to a 3.23-fold (multivariate OR, 95% CI: 1.53–6.80, *P* < 0.001) higher risk of SARS-CoV-2 positivity compared to negative patients. Obesity was not a significant risk factor for SARS-CoV-2 positivity (1.86-fold, multivariate OR, 95% CI: 0.53–6.56, *P*=0.334).

Finally, six patients were intubated, two died, and one case had multiorgan dysfunction syndrome (MODS). The case-fatality rate was 1.6%.

## 4. Discussion

The main result of this study is the clear importance of family member contact in the risk of positive SARS-CoV-2 tests in the pediatric population. It was also found that CO-RADS was associated with SARS-CoV-2 positivity in the Mexican pediatric population and that the clinical and laboratory data differed from other reports worldwide. The mortality rate in the population studied was higher compared to reports from other countries.

This is the first study to show that the main variable associated with SARS-CoV-2 positivity in the Mexican hospitalized pediatric population is contact with a SARS-CoV-2 positive family member, a situation that can be explained by the fact that during the period in which the study was conducted, social distancing policies prevented this population from attending school [30]. However, we do not have enough information to estimate the direction of transmission since it is not possible to know who was the index case. Despite this, it is important to emphasize that children are considered to have a low risk of transmitting the disease and tend to have low viral loads compared to adults [31].

Our study found no difference in the gender of children with positive and negative SARS-CoV-2 tests, which agrees with other reports [2]. The age of confirmed patients was different from the study by Eastin and Eastin [32] in the Chinese population but similar to the study by Graff et al. [33] in the Italian population. The difference in age among studies may be due to the different criteria of inclusion between each study; while the study by Dong was performed in the general population, the study of Graff, like ours, was carried out in the hospitalized population. Like other studies, obesity was more frequent in the group of SARS-CoV-2 positive patients [33]. In this study, the comorbidities studied did not increase the risk of a positive test, which agrees with other observational studies [2]. However, a large proportion of those in this cohort who were hospitalized had comorbidities, suggesting that comorbidities in the pediatric population may be associated with greater severity of SARS-CoV-2 infection, as other studies have found in hospitalized populations [32].

The CO-RADS scale has not been validated in the pediatric population [34]; however, it has been found that patients younger than 15 years are less susceptible to alterations suggestive of SARS-CoV-2 infection according to the CO-RADS scale [35]. Additionally, another report found that the CO-RADS scale is not associated with the severity of SARS-CoV-2 infection [6]. In this work, we found a statistically significant association between the CO-RADS scale and positive cases. Differences in the interpretations of CO-RADS results may occur due to age-related differences in the distribution of lesions in SARS-CoV-2 infected patients [36]. However, it is important to consider that the population included in this study consisted of patients with moderate to severe disease severity, and given the small population, it was not possible to include severity in the multivariate model. Furthermore, there is insufficient information to support the use of CO-RADS as a diagnostic tool for SARS-CoV-2 infection. Therefore, the use of CT should be reserved as a diagnostic tool in the case of multiple primary lung diseases in SARS-CoV-2 infection [37].

The distribution of symptoms between positive cases and reference values obtained from the meta-analysis by Cui [27], which consisted of a higher proportion of the pediatric population from the United States of America and China [27], showed that the Mexican population shows different clinical behavior, with a higher frequency of fever, sore throats, rhinorrhea, tachypnea, diarrhea, and vomiting. The observed difference may be explained by the fact that our cohort consisted mainly of hospitalized patients, and it can be assumed that they were sicker and therefore more symptomatic. In addition, the Mexican pediatric population has a high prevalence of metabolic syndrome, which has been associated with elevated ACE2 expression in the cell membrane, which may increase the virulence of SARS-CoV-2 [38]. Fever and fatigue were two of the symptoms that could help differentiate positive cases. Furthermore, in the multivariate model, fever and fatigue statistically significantly increased the risk of being a positive case.

Case-fatality in the pediatric population (1.6%) was low with respect to hospitalized Mexican adult populations (24%) [39]. Multiple hypotheses have been proposed to explain the lower probability of developing a severe case of SARS-CoV-2 infection in children and adolescents; among the main ones are the lower expression of ACE2 and less polarization towards inflammatory phenotypes of monocytes in children compared to adults [40]. However, case-fatality among the pediatric population in our study is very high compared to that recorded in other parts of the world and is very similar to that recorded in national databases in Mexico [41]. Therefore, there is sufficient evidence to support the hypothesis that the pediatric population of Mexico has one of the highest mortality rates in the world. This situation can be explained by the high prevalence in the Mexican pediatric population of lifestyle habits that lead to becoming overweight and having metabolic syndrome, among other diseases. These conditions favor proinflammatory environments and increased expression of ACE2, with a higher probability of developing a severe case [42]. Unfortunately, we did not have the necessary information to evaluate these and other comorbidities that could help us to understand the high mortality of children and adolescents in Mexico. The assessment of obesity, vaccination scheme completion, ABO groups, and lymphocyte and leukocyte levels was not useful in the identification of SARS-CoV-2 positive cases.

There are several strengths of this study. First, the sample consists of hospitalized patients who underwent clinical and laboratory follow-up during their stay, while most of the studies published in Mexico consist of national database analyses, in which there are many variables that can act as confounders. Secondly, the sample size is one of the largest in Latin America. Third, multivariate models were fitted to assess the effect of other confounding variables.

However, this study also has some limitations. First, it is a retrospective study. Second, the cohort consisted only of Mexicans treated in Mexico City, so the results cannot be generalized to other populations. Third, only patients hospitalized at one center were included, which may increase the probability of selection bias. Fourth, we have insufficient data to estimate severity indexes. Fifth, information regarding the number of household members was not available for inclusion in the PSM model. Sixth, children with milder SARS-CoV-2 infections may have not been included in this study, among them children hospitalized with SARS-CoV-2 infections instead of directly because of SARS-CoV-2 infection.

In conclusion, the present study found that the presence of contact with a family member who was positive for SARS-CoV-2 with suggestive symptoms such as fever and fatigue should be considered factors associated with a positive confirmatory RT-PCR for SARS-CoV-2.

New studies with larger numbers of patients should focus on the pathophysiological mechanisms of COVID-19 in children and adolescents, the difference between pediatric age groups in CT imaging presentation in COVID-19, and ACE-2 polymorphisms and other proteins associated with COVID-19 virulence in the Mexican pediatric population. In addition, new models should consider the number of days from symptom onset and the number of household members.

## Figures and Tables

**Table 1:** Comparison of demographics, clinical features, comorbidities, and hematological parameters between SARS-CoV-2 positive and negative pediatric patients.

Variable	SARS-CoV-2 positive, *n* = 36	SARS-CoV-2 negative, *n* = 85	*P*value
*Demographics*			
Sex (male)	16 (44.4%)	49 (57.6%)	0.1
Age (years)			
<5	12 (33.3%)	43 (56.6%)	**0.006**
6 to 10	6 (16.7%)	17 (20.0%)	
11 to 15	16 (44.4%)	13 (15.3%)	
15 to 17	2 (5.6%)	12 (14.1%)	
Contact with a confirmed case	8 (22.2%)	6 (7.1%)	**0.01**

*Clinical features*			
Hospitalization (>1 day)	17 (47.2%)	38 (44.7%)	0.7
Comorbidities			
Asthma	3 (8.3%)	4 (4.7%)	0.4
Immunosuppression	5 (13.9%)	15 (17.6%)	0.6
Cancer	5 (13.9%)	24 (28.2%)	0.09
Hypertension	1 (2.8%)	1 (1.2%)	0.5
Obesity	5 (13.9%)	3 (3.5%)	**0.03**
Allergies	3 (8.3%)	9 (10.6%)	0.7
CO-RADS	*n* = 14	*n* = 14	
1	1 (7.1%)	8 (57.1%)	**0.003** ^**∗∗∗∗**^
2	2 (14.3%)	0 (0.0%)	
3	1 (7.1%)	0 (0.0%)	
4	0 (0.0%)	2 (14.3%)	
5	10 (71.4%)	4 (28.6%)	
Completed vaccination scheme^*∗*^	*n* = 15	*n* = 37	
No	0 (0.0%)	8 (21.6%)	0.1
Incomplete	4 (26.7%)	9 (24.3%)	
Complete	11 (73.3%)	20 (54.1%)	

*Hematological parameters*			
ABO blood group	*n* = 13	*n* = 24	
O	9 (69.2%)	18 (75.0%)	0.9
A	2 (15.4%)	3 (12.5%)	
B	2 (15.4%)	3 (12.5%)	
Lymphocytes	*n* = 18	*n* = 45	
Normal^*∗∗*^	4 (22.2%)	13 (28.9%)	0.6
Low	13 (72.2%)	27 (60.0%)	
Hight	1 (5.6%)	5 (11.1%)	
Leukocytes	*n* = 18	*n* = 45	
Normal^*∗∗∗*^	9 (50.0%)	14 (31.1%)	0.3
Low	7 (38.9%)	21 (46.7%)	
Hight	2 (11.1%)	10 (22.2%)	

^*∗*^Complete Mexican vaccination scheme.^*∗∗*^Normal ranges by age and gender: 6 months–<3 years old: male (2.34–5.45 × 10^3^/mcL) and female (2.34–6.44 × 10^3^/mcL); 3–<6 years old: male and female (1.6–5.3 × 10^3^/mcL); 6–<12 years old: male and female (1.4–3.9 × 10^3^/mcL); and ≥12 years old: male and female (1–3.2 × 10^3^/mcL).^*∗∗∗*^Normal ranges by age and gender: 6 months–<3 years old: male (7.73–13.12 × 10^3^/mcL) and female (7.05–12.98 × 10^3^/mcL; 3–<6 years old: male and female (4.4–12.9 × 10^3^/mcL); and ≥6 years old: male and female (3.8–10.4 × 10^3^/mcL).^*∗∗∗∗*^Bootstrap *P*value. Significant *P*values are bolded.

**Table 2:** Comparison of symptoms between SARS-CoV-2 positive and negative pediatric patients.

Symptom	SARS-CoV-2 positive, *n* = 36	SARS-CoV-2 negative, *n* = 85	*P*value
Dyspnoea	8 (22.2%)	27 (31.8%)	0.2
Headache	11 (30.6%)	27 (31.8%)	0.8
Fever^*∗*^	33 (91.7%)	60 (70.6%)	**0.01**
Cough	17 (47.2%)	39 (45.9%)	0.8
Sore throat	9 (25.0%)	17 (20.0%)	0.5
Tachycardia^*∗∗*^	4 (11.1%)	12 (14.1%)	0.6
Rhinorrhea	11 (30.6%)	31 (36.5%)	0.5
Nasal congestion	2 (5.6%)	0 (0.0%)	0.1
Tachypnea^*∗∗∗*^	7 (19.4%)	15 (17.6%)	0.8
Diarrhea	7 (19.4%)	20 (23.5%)	0.6
Vomiting	13 (15.3%)	4 (11.1%)	0.5
Myalgia	7 (19.4%)	18 (21.2%)	0.8
Fatigue	8 (22.2%)	7 (8.2%)	**0.03**
Hypoemia	3 (8.3%)	2 (2.4%)	0.1
Chest pain	3 (8.3%)	2 (2.4%)	0.1

^*∗*^ >38.2 Celsius at the level of the axilla, ranges do not vary with age.^*∗∗*^Tachycardia cutoff: 1–<12 months old (>190 beats/min); 1–<3 years old (>140 beats/min); 3–<6 years old (> 120 beats/min); 6–11 years old (>118 beats/min); and ≥12 years old (>100 beats/min).^*∗∗∗*^Tachypnea cutoff: <1 year old (>53 breaths/min); 1–<3 years old (>37 breaths/min); 3–6 years old (>28 breaths/min); 6–11 years old (>25 breaths/min); and ≥ 12 years old (>20 m breaths/min). Significant *P*values are bolded.

**Table 3:** Comparison of symptoms of positive patients with the percentages obtained in the meta-analysis of Cui (2019).

Symptom	SARS-CoV-2 positive, *n* = 36	Meta-analysis values^*∗*^*N* = 5829	*P*Value
Dyspnoea	8 (22.2%)	NA	NA
Headache	11 (30.6%)	NA	NA
Fever	33 (91.7%)	51%	**<0.001**
Cough	17 (47.2%)	41%	0.4
Sore throat	9 (25.0%)	16%	**<0.001**
Tachycardia	4 (11.1%)	12%	1
Rhinorrhea	11 (30.6%)	14%	**0.01**
Nasal congestion	2 (5.6%)	17%	0.07
Tachypnea	7 (19.4%)	9%	**0.03**
Diarrhea	7 (19.4%)	8%	**0.02**
Vomiting	13 (15.3%)	7%	**<0.001**
Myalgia	7 (19.4%)	12%	0.1
Fatigue	8 (22.2%)	12%	0.07
Hypoemia	3 (8.3%)	3%	0.09
Chest pain	3 (8.3%)	3%	0.09

^*∗*^Taken from J Med Virol. 2021; 93: 1057–1069 (15). Significant *P*values are bolded; NA, no applicable.

## Data Availability

The data sets used to support the findings of this study are available from the corresponding author on reasonable request.
